# Investigation the role of contrast on habituation and sensitisation effects in peripheral areas of graphical user interfaces

**DOI:** 10.1038/s41598-022-16284-2

**Published:** 2022-09-10

**Authors:** Anna Lewandowska, Malwina Dziśko, Jaroslaw Jankowski

**Affiliations:** West Pomeranian University of Technology, Szczecin, Żołnierska 49, 71-210 Szczecin, Poland

**Keywords:** Computer science, Information technology, Human behaviour

## Abstract

Graphical user interfaces are designed so that the most important elements are usually located in the central part of the screen, where they catch the user’s attention. However, there are situations where it is necessary to attract the user’s attention to make him/her notice, e.g., a critical alert, which is customarily displayed in the peripheral area so as not to interact with the main content. Therefore, our focus is to deliver an increased visibility of content in the peripheral area of the display in a non-intrusive way. Thus, the main purpose of this work is to analyze the visibility of the stimulus (in the form of colored discs), displayed in the peripheral area of a screen, which distracts users from the central part of the interface. The habituation and sensitization effects were considered to study which parameters catch and hold the user’s attention, despite the length of their interaction with the system. The experiments performed indicated how the parameters should be set to reduce the habituation effect without the need to use content with the highest visual intensity. The results showed that a high visual intensity is not necessarily needed for the best impact. A medium contrast level, a horizontal or vertical display localization, and a flashing frequency of 2 Hz are sufficient to obtain the best visibility in the peripheral area. In the case of critical alerts and the need for short-term intensive stimuli, it is worth highlighting these with high contrast. This configuration should be the most effective if it is not a continuous operation. However, they can cause unnecessary irritation or even cognitive load for more extended usage.

## Introduction

Graphical user interfaces are an indispensable bridge between human beings and the surrounding environment, from IT (Information Technology), cash machines, and sales systems to specialized equipment related to specific professions such as surgery, aviation, and the manufacturing industry^1,2^. A large part of a graphical user interface is created so that the information relevant from the work perspective focuses the user’s attention on the central part of the interface, meaning that what is displayed in the peripheral area becomes much less noticeable to the user. The problem is that, nowadays, a user working with various interfaces (from websites to interfaces with games) is inundated with loads of unnecessary alerts and information^3^. Such alerts displayed on the user’s monitor (often in the peripheral area) very often fall under a habituation effect that causes them to be ignored^4^. Unfortunately, such actions may have a negative effect in the case of the user’s later work with the interfaces when a very important alert is displayed (e.g., regarding the interface on the surgeon’s monitor during an operation, which could be life-threatening), and the user will bypass it out of habit^5,6^. A similar problem affects people working with graphical user interfaces on a monitor that is set in the peripheral area of their eyesight, e.g., in dentistry. In that case, during, e.g., root canal treatment, a stomatologist focuses his/her attention on the patient but at the same time controls the effect of a treatment (probe depth in the root canal) on the monitor located in his/her peripheral area.

**Figure 1:**
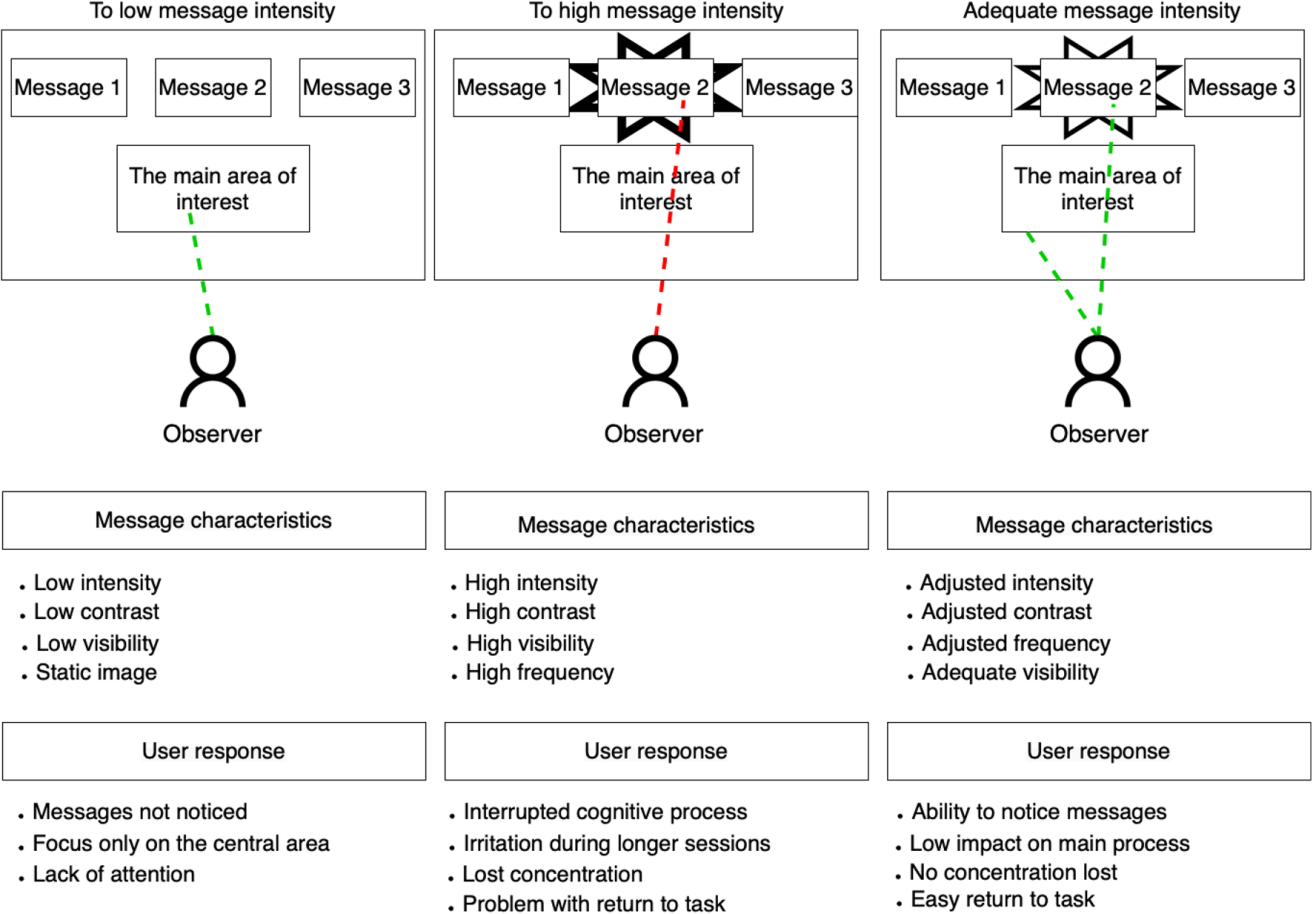
Interface variants with different visual intensities of presented messages in peripheral areas and the impact on the system user for low (Left), high (Middle), and target goal (Right)—adequate message intensity.

Response within the interface can be decreased due to the habituation effect based on stimuli repetition with an associated lack of reinforcement or importance for the target user.^7^. The stimulus will be ignored or an attention drop will be observed if it has lower significance for the receiver. A similar effect to habituation is based on sensory adaptation, but sensory adaptation is a result of a sense organ limited reaction to repeated stimuli^8^. While it cannot be controlled, the habituation can be controlled consciously^9^. Sensitization is the opposite effect of habituation, characterized by an increased response to repeated stimuli because of their significance to a receiver^7^. Even with a lower intensity of stimuli, the response will be continued until its significance is observed. Both habituation and sensitization processes are automatic adaptations to the environment. They are a form of simple non-associative learning without any reinforcement or from the environment^8^. In terms of user interfaces, the habituation effect results in a lower response to messages within the interface including visual or textual messages^10^. An increase in sensitization level could help deliver important messages presented within the interface during a user session (see Fig. 1).

In attempts to increase sensitization to attract user attention, techniques based on visual elements can be used. Unfortunately, they often lead to the perceived intrusiveness of the interface^11^. Lowering the intensity of visual elements makes the user interface more friendly, especially during more extended usage. Still, a total lowering of the component’s intensity inhibits the passing of important messages that are not perceived. The problem of habituation thus returns.

Therefore, questions arise: How can the habituation effect toward sensitization be reduced? How can the user’s attention be held without its having a negative impact on the user’s task? When the stimulus obtains user attention during a session without disturbing user concentration on the primary task, are there any thresholds of stimulus parameters, such as contrast, location, or flickering frequency, when the stimulus obtains user attention during a session without disturbing user concentration on the primary task? The answers to these questions are our motivation for this paper.

**Figure 2:**
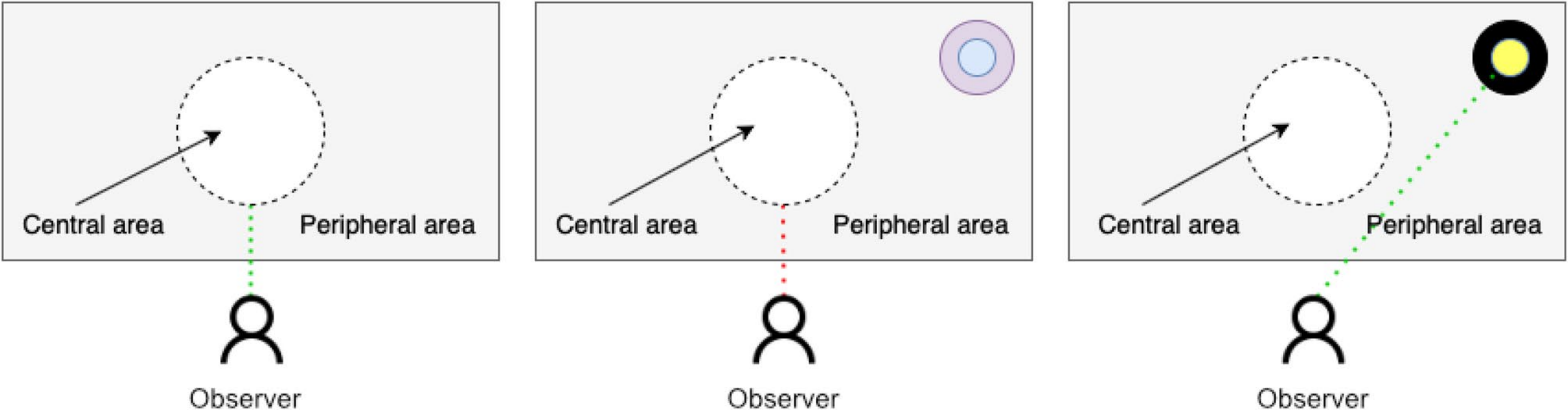
The visibility of the stimulus on the display at different levels of contrast. Left side: no stimulus, the correct focus of the observer in the central part of the display. Center: low contrast of the stimulus, unnoticeable by the observer, due to its location and wrong contrast selection. Right side: correspondingly higher contrast of the stimulus noticeable by the observer in a part of the display other than the center.

Considering these problems of graphic interface design, which relate to ignoring potential peripheral vision areas due to habituation, we investigate the ability to increase the efficiency of these areas while working with the interface by adjusting the visual contrast and the relevant frequencies. The structure of the human eye translates into the visibility of the contrast, which is significant in the process of interface design^12^. Because the cones are perfect for good lighting, especially in the central part^13^, the contrast used to create interface elements (central part) or stimuli that are intended to distract the user should have low contrast. The more we want to move the interface elements away from the central region towards the periphery, the higher the contrast these elements should be. This is because rods allow an observer to perceive peripheral and dimly lit elements, which do not produce images as accurate as cones do^13^, see Fig. 2. Therefore, in our research, we used discs with different flicker frequencies. However, the discs were in a permanent place. We decided to use discs that are not dynamic (do not move) for the test because specific elements that make up the graphical user interface (e.g., alerts) are displayed in a strictly defined area for a given graphical interface. We did not want to break this rule.

Our main aim was to search for effective ways of increasing the visibility of visual objects in the peripheral area without having a negative impact on the user’s task. Therefore, our priority is to point out how to avoid the habituation effect and achieve sensitization with low visual intensity represented, by contrast, frequency flickering and the localization of stimuli.

The paper is organized as follows First, we introduce the state-of-the-art in the Related Work section. The conceptual framework and subjective experiment (which explains in detail the subjective experimental procedure) are presented in the Methods section. The results and analyses can be found in the Results section. A description of potential development directions concerning the state-of-the-art can be found in the Discussion section. Finally, we concluded the paper in the Conclusions section.

## Related work

Apart from providing fast access to information, current technologies should be designed to support human attentional processes, on which they impose further strain. The analysis of issues related to the design of systems capable of such support was introduced in^14^. When designing graphical user interfaces, in the context of human attention, especially in the peripheral areas of human vision, knowledge about the structure of the human eye is crucial^15^. In particular, it concerns the distribution of photoreceptors in the retina, which influence the lower resolution of the image seen in the peripheral area and thus deteriorates color vision and contrast sensitivity. The habituation effect complements the considerations concerning the human attention process in the peripheral areas of vision.

### Exploration of the visibility in a peripheral area of graphical user interface

Previous research shows that displaying the disc in the peripheral area of the interface with an appropriate set of parameters diverts attention from the central part of the interface and focuses it on the displayed disc^4,16–18^. The literature indicates that the most common approach to distracting the user from the central part of the interface is to display a single flashing disc for a split second^19,20^. However, it is not always possible to notice the displayed disc, especially in the case of full focus on the task being performed^21^. Such a disc is often noticed accidentally, or by deliberately redirecting one’s eyes towards the disc^21,22^. Another approach described in the literature is to display discs that are not modulated in time. These discs are displayed continuously^19,20,23^. The consequence of that approach may be the observer getting used to the displayed disc, as it may become an integral part of the interface to the observer and no longer attract attention to itself.

A different approach was demonstrated in^22^. In this paper, the peripheral vision was examined using physical elements that vibrated and were outside the monitor frame. It turned out that the movement of the elements caused a focus on the vibrating element, especially when they were moving at the top of the monitor. Unfortunately, they did not discuss the frequency of vibrations of the distracting stimulus, nor its contrast between the color of the stimulus and the background. Another important factor influence on peripheral vision, i.e., color, was discussed in^17,18,23,24^. The authors focused on making the best visibility of colors in the peripheral area by employing a continuous disc that changed color and contrast. In the paper, it was shown that the sensitivity to changes in the peripheral area drops more sharply with a color change from red to green than with a color change from yellow to blue. Increasing the contrast drew the observer’s attention to the peripheral area. However, the subject concerning the contrast threshold, where the object is visible but with a small amount of intrusiveness, was not a subject of discussion.

A similar problem was indicated in^16,25,26^, where the increase in viewing angles was reported as causing the peripheral vision to deteriorate. These works are supported by^4^. The authors showed that the greater the observer’s viewing angle is, the worse the observer’s perception is of the color and contrast of the displayed elements. Thus, in^4^, they recommend that, as the viewing angle increases, the contrast level of the displayed element should be increased. However, the contrast threshold at which increasing the insensitivity of attributes generates visibility without affecting the user’s health is unclear. The authors analyzed both the peripheral and central areas. After the experiment, the observers completed a survey about the information displayed. It turned out that most of the correct answers appeared when the question was about the information displayed in the peripheral area. This leads to the conclusion that, if items in the peripheral area are displayed appropriately, they are likely to attract the attention of observers better than the items shown in the central area. However, the authors did not indicate detailed parameters of the displayed information in the peripheral area (such as the color or the contrast), which may be necessary from the perspective of graphic interface design. The approach differs from our conception, as here, the people watched the screen freely, unlike our experiment where the attention had to be concentrated on the central part of the screen.

Despite extensive research on the topic of graphical user interfaces in the peripheral range, there remains a gap regarding the effectiveness of distracting the attention of a user entirely focused on a task being performed using the central part of the screen. Performance of the interface can be improved by the redirection of user attention from the main area to the peripheral section with messages necessary for the performed task, e.g., blood pressure during surgery. While low visual intensity leads to unnoticed important messages, contemporaneously, high visual intensity results in irritation and negative consequences for the main cognitive process. Previous works related to peripheral vision associated with graphical user interfaces do not provide parameterized values of stimuli placed in the peripheral area that can be used with real user interfaces. There are some generalized studies indirectly related to user interfaces that concern short-flash (single flash) and continuous (constant frequency) disc images^19,20^. Still, the parameters do not apply to computer systems because of their high frequencies.

Our research extends the studies above by analyzing the flashing frequencies and the position variants of discs where they are displayed, especially when the contrast of a stimulus is changed from a low level to medium and high levels. The selected and analyzed disc positions were dictated by a practical framework: critical alerts and other messages are typically displayed in peripheral areas.

### The habituation and sensitization influence on communication within user interfaces

The habituation effect, defined as “a behavioral response decrement that results from repeated stimulation and that does not involve sensory adaptation/sensory fatigue or motor fatigue”^8^, has been widely analyzed among humans, animals, and even plants^27^. Habituation is understood as a learning process leading to content being ignored and additional knowledge failing to be delivered^28^. Apart from psychological or biological studies, habituation has been analyzed from the perspective of technical systems and user interfaces. Early-stage research was based on various parameters used to gather general knowledge about the habituation effect. For example, adaptation to peripheral flicker was studied with the use of frequencies challenging to implement due to usability factors^29^. Together with habituation, a sensitization effect was explored and identified as part of a dual process that assumes that behavior is the result of summarized habituation and sensitization^30^. Due to sensitization, increments in responsiveness on early contacts with visual stimuli were observed, together with decay over time^31^.

Recently, studies have focused on computer systems and the role of habituation in the system and interface design. Most of them have been based on displaying sets of images with different setups or flashing with different frequencies. They require different empirical setups and realistic content to have more practical goals. Habituation has also been examined from the perspective of web interfaces and marketing as a form of banner blindness^32^. One study showed that visual marketing content is ignored unconsciously due to the habituation effect. It was also observed in textual marketing content^33^. Visual marketing content increases perceived workload and, as a result, hinders visual search^10^. Banner blindness was examined with the use of eye-tracking^34^ with a particular focus on content location and user tasks. These observed phenomena create a need for attracting user attention through several other techniques, such as increased visual intensity, animations, flickering effects, verbal communication with varying intensity^35^, gradual approaches^36^, and manipulation of the information type of the content^37^.

Another direction has focused on habituation concerning security warnings, which is summarized in a review paper^38^. Studies in this area analyzed eye-movement-based memory and showed that habituation is identified after a few exposures to warning messages and develops fast with subsequent iterations^39^. An fMRI-based study showed that habituation is more intensive concerning security warnings than to any other type of image^40^. Authors emphasize the need for interface designs less susceptible to habituation and habituation-resistant security warnings.

Other work analyzed user-interface modifications to attract users’ attention to the content affecting taken decisions^41^. The authors used inhibitive attractors and proved that they reduce the number of software installations with suspicious components. Attractors improved security and decreased the proportion of users selecting the less safe option. Another paper studied arousal strength associated with signal words and icons that commonly appear in exception messages^42^. Another study focused on security warnings in mobile applications with the use of longitudinal fMRI approaches and security warnings ^43^.

Earlier studies have delivered general directions and guidelines for which techniques should be used to overcome habituation within user interfaces, for example, polymorphic warning messages^44^. Recent work has also focused on visual effects added to privacy notes^45^.

The impact of different frequencies on habituation has been analyzed. Still, the question of what the contrast between used colors in the flickering effect should be has remained an issue. A study with contrast variations compared the impact of white text on a black background versus black and grey combinations^39^. In another study, authors considered ANSI guidance for warnings with high contrast font colors and backgrounds^41,46^; however, only yellow text on a black background to draw attention to the salient field was used.

The question of what contrast is sufficient to attract attention and reduce habituation has not yet been answered. Aggressive techniques can be considered intrusive. They increase the irritation level and cause negative feedback towards the message sender^11^. While intensive visual elements can be helpful for short-term usage, they can decrease user experience during more prolonged contact with the interface, such as surgery or medical treatment, games, or video sessions. We aim to search for the lowest possible intensity and the highest potential to draw attention.

### Contrast in the peripheral area

When considering the subject of user interfaces and attracting the user’s attention, it is impossible to ignore one more key parameter, such as a contrast between the color of a displayed stimulus and its background color. The human eye perceives more contrasting elements in everyday life than those whose contrast is low. This is because they are more distinct; often, their color is more saturated. The high contrast of various stimuli causes the human sight to be subconsciously redirected to these stimuli (it results from the structure of the eye). The same applies to the perception of contrast in the peripheral areas. Previous studies have shown that the human eye perceives low contrast and high contrast stimuli differently in the peripheral area. In most of the literature, the topic of the perception of chromatic and achromatic stimuli has been studied^23^. For this purpose, the paper’s authors created experiments to display stimuli in the peripheral area. Mostly these were stimuli created using the Gabor pattern (stripes). Then they displayed these stimuli, increasing the frequency of the fringes at such an angular distance that the participants of the experiments could see them from the corner of their eyes without having to redirect their eyesight. After the research, it turned out that the higher the frequency of the bands, which resulted in the increased contrast of the stimulus, the better the observer perceives the stimulus, and with the decrease in frequency, its sensitivity decreases.

**Figure 3:**
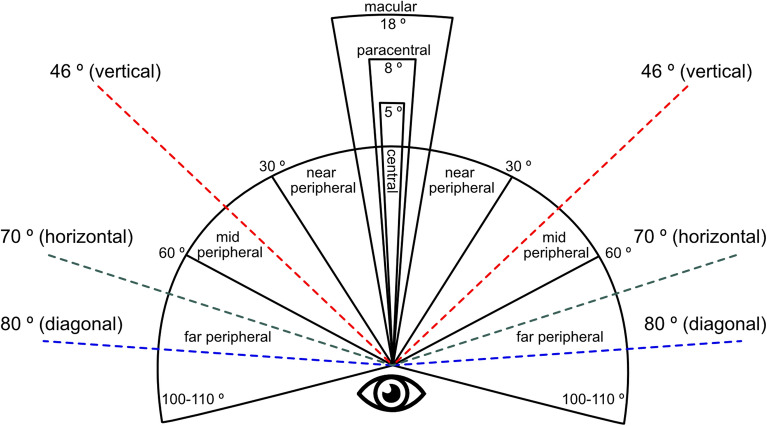
The scope of human vision^48^. The dashed lines represent the angular distances we use in subjective experiments (“Subjective experiment” section).

Figure 3 shows the human vision range and the sharpness that the human eye can detect in a given area (for monocular vision). At an angular distance of 5°, objects are seen by humans with the most excellent sharpness. The range of view up to an angular distance of 18° is slightly less sharp but still can see objects accurately. As people move away from this angular distance, towards 30°, human visual acuity decreases until it reaches the peripheral field of view ranging from 30° to 110°. Center vision defines the area in which a person can achieve visual acuity. The selected values correspond to the natural angles distributed along the edges of the screen. It should also be mentioned that the scope of the human peripheral vision field also depends on biological factors. These factors include, among others, the width of the distance between the eyes and the location of the eyeballs in the sockets^47^.

The peripheral area covers the field of view on which a person is not focused at the moment^49^. Thus, an essential aspect in studying the role of the contrast of a stimulus located in the peripheral area and its impact on human habituation and sensitization is the human eye’s sensitivity to contrast. As indicated in the literature^49,50^, the contrast sensitivity function determines the sensitivity of human eyesight to contrast, assuming constant frequencies.

## Results

The experimental results were subjected to statistical analysis in three areas: (1) the effectiveness of attracting the user’s attention to the peripheral area while focusing on the cognitive task located in the center of the screen, (2) the habituation and sensitization effect, and (3) the impact of localization and flickering frequency on visibility in the peripheral area. By the term ***visibility***, we refer to the percentage of users among all participants noticing displayed discs.

### Observers screening through eye-tracking (ET) data analysis

Before starting the analysis to obtain reliable results, it is necessary to verify the correctness of the collected data. In the case of our research, it was essential to ensure that, during the study, users focused their attention on the screen and not on the flashing discs in the periphery.

**Figure 4:**
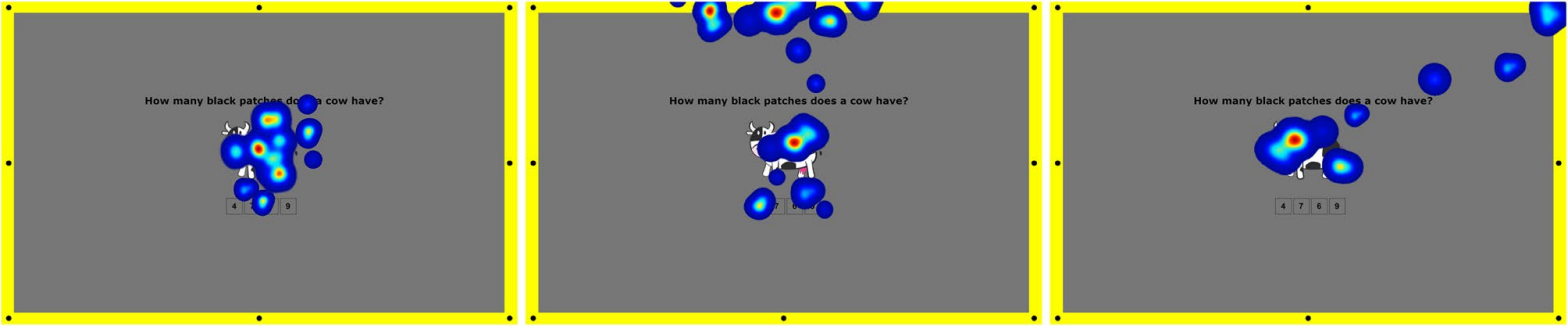
The course of the experiment and the corresponding heat maps. (left) The observer focuses strictly on a given cognitive task: the experiment was conducted properly. (center and right) The user fixed his sight on the stimuli in the peripheral space instead of focusing purely on the cognitive task: the experiment was conducted improperly.

Therefore, the first step after a series of perceptual experiments was to validate the recorded data based on the signal obtained from the eye tracker. This enabled us to recognize and screen the people who deliberately redirected their eyes to the displayed disc. An example of such a situation is depicted in Fig. 4. To reject the respondents that, during the experiment, instead of concentrating on cognitive tasks, deliberately focused on discs displayed on the screen’s periphery, we used two criteria. The first one involved the measurement of time spent on color discs if the respondent redirected his/her eyesight from cognitive tasks to the periphery area. If the time spent watching discs was longer than 0.5 s, the respondent’s data were rejected. According to documentation used in the experiment eye-tracker glasses^51^, a time of 5 s was reported as the minimal time of fixation duration. The second criterion identified the situation when the respondent’s focus was on the edge of the screen rather than its central part. As a result of the investigation, 2 of 60 were excluded from further analysis because of an inappropriate performance, so the data collected from 58 participants were finally included in the study.

### Human attention in the peripheral area

As increasing user attention in the peripheral area of the display in a non-intrusive way is our focus, one of the main aims of the research was to analyze the visibility of the stimulus (discs), displayed in the peripheral area of a screen, that distracts users from the cognitive task given in a central part of the interface.

**Figure 5:**
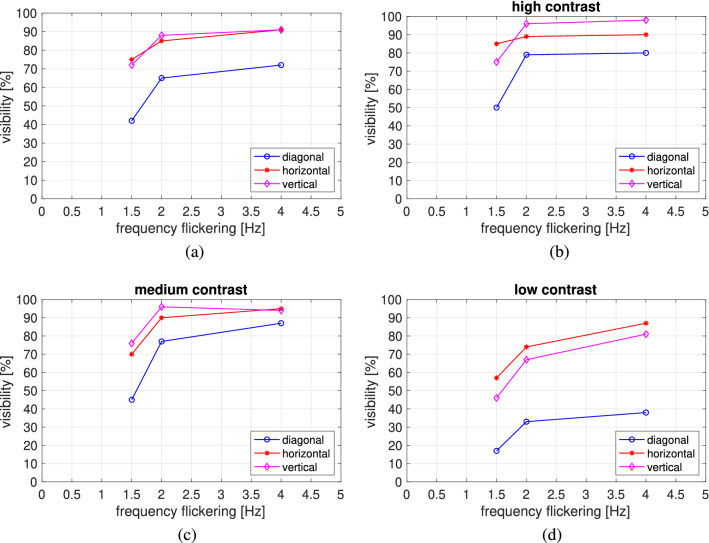
Disk visibility is represented as the percentage of users noticing stimuli given for (**a**) a general approach (mean contrast), (**b**) a high contrast, (**c**) a medium contrast, and (**d**) a low contrast of disc color and background, given for three different frequencies of flickering: 1.5 Hz, 2 Hz, and 4 Hz and for three different angular distances: horizontal (46 deg), vertical (70 deg), and diagonal (80 deg).

The data, abbreviated as disc visibility, represent the number of cases per observer, given as a percentage, where the stimulus, i.e., its location, has been correctly identified. We analyzed the visibility of the discs for different locations and frequency setups. The obtained results are depicted in Fig. 5. The analysis showed that, together with the higher frequency of the flashing disc and the smaller the angular distance, the visibility of the disc grows. In a general approach, the acceptable level of visibility above 80%, defined by the percentage of users among all participants noticing displayed discs, was reached only for vertical and horizontal locations starting at a frequency equal to 2 Hz and with a medium contrast. The rest of the setups attracted users’ attention below 80%.

#### Contrast analysis

To analyze the effects of contrast, we chose three different contrast levels: low (violet-blue: F 1.4:1), medium (black-gray: P 7.6:1), and high (black-yellow: P 19.6:1). The results are depicted in Fig. 5b–d, respectively.

*With high contrast* (19:1) (see Fig 5b), a disc visibility above 90% was reached with vertical and horizontal locations at frequencies starting from 2 Hz. Additionally, the only setup with a low frequency that reached visibility close to 90% was the vertical one. The discs in the rest of the setups with 1.5 Hz were noticeable below 80%. In the case of diagonal locations, the visibility level of 80% was achieved at 2 Hz and 4 Hz. However, it was much lower than those at the horizontal and vertical locations.

*With medium contrast* (7.6:1) (see Fig. 5c), as before, a disc visibility close to 90% was achieved with vertical and horizontal locations at frequencies starting from 2 Hz. In this case, however, a frequency of 1.5 Hz rarely caught the user’s attention when compared to high contrast, e.g., at a level of about 70%. In the case of a diagonal location, a level of visibility around 80% appeared only from the flashing frequency at 2 Hz.

*With a low contrast* (1.4:1) (see Fig. 5d), the difference between the visibility of flashing objects in the peripheral area in the diagonal location turned out to be much higher compared to the horizontal and vertical ones. Accordingly, at all frequency levels, the visibility in the diagonal position was approximately two times lower than in the other locations. The best-perceived location was horizontal, but a level above 80% was achieved (similarly to the horizontal location) only at 4 Hz.

*Summary*. Summarizing the results, an important issue was that, in the case of a high or medium contrast, the locations where the vast majority of users noticed flashing discs turned out to be vertical and horizontal, with a result above 90%. In the case of vertical and horizontal locations with a high contrast, it was even 98%, which, in the case where one needs to attract attention quickly and in the short term, seems to be a very good solution. The situation concerned flashing frequencies from 2 Hz. In the case of a diagonal location, only a level of 80% was obtained, starting from a frequency of 2 Hz.

By reducing the contrast to a low level, the only locations to consider are vertical and horizontal locations with a flashing frequency of only 4 Hz. However, they are only noticeable at the 80% level. The diagonal location, obtaining a result twice lower than the others, seems not worth attention and further consideration.

### The significant difference and the effect size: location and flashing frequency impact on visibility in the peripheral area

To emphasize and confirm the experimental results, the effect size (ES) and variance analysis between the compared groups with different setups (levels of contrast, flashing frequencies, and angular distances) were taken into account.

#### One factor-setup analysis

**Figure 6:**
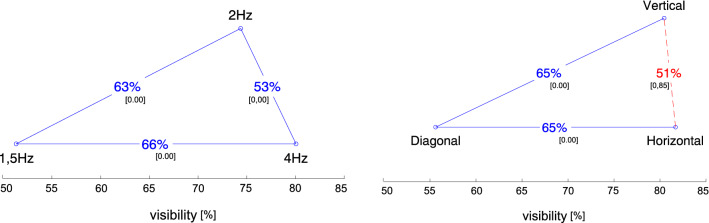
Average visibility results. The ranking shows the relationship between the groups with respect to frequency (Left) and in terms of angular distance (Right). Each blue circle represents a tested condition and they are ordered according to their ranking, with the least preferred condition on the left. The x-axis represents the rating of each condition, expressed as the mean number of votes, i. e. mean visibility of the disks with given setup. The percentages indicate the probability that an average observer will regard the condition on the right as better than the condition on the left. If the line connecting two conditions are red and dashed, there is no statistical difference between this pair of conditions ($H_0$ could not be rejected at 0.05 level). The small-font number in square brackets indicates the *p* value of the *t* test between both conditions (without compensation for multiple comparisons).

First we checked a condition ranking without a contrast breakdown for location and frequency (see Fig. 6). One-way ANOVA was performed first, indicating significant differences between the conditions of frequency ($p\,\hbox{value} = 5.999e^{-57}$, $F(2) = 133.87$) and location ($p\,\hbox{value} = 1.693e^{-66}$, $F(2) = 157.5$). To confirm the differences between the conditions of a given setup, we then ran posthoc tests, utilizing a *t* test between the closest and more distant adjacent conditions in the visibility ranking.

We found a statistically significant difference between the corresponding levels of both frequencies (Left) and location (Right), respectively. To increase the readability of the results, we include detailed information about the analysis factors in Table 1. The most visible elements had a flickering frequency of 4 Hz; the least visible were those flashing at 1.5 Hz ($U_3 = 66\%$ means that more than 6 out of 10 users spotted elements flickering at 4 Hz; at 1.5 Hz, less than 4 out of 10 did so). A statistically significant difference was found in the means between the pair of frequencies 2 Hz and 1.5 Hz with effect size (ES) *d*-Cohen $= 0.3292$ ($U_3 = 63\%$ means that more than 6 out of 10 users spotted elements flickering at 2 Hz; at 1.5 Hz, less than 4 out of 10 did so). The mean visibility with a frequency of 2 Hz was less than that with a frequency of 4 Hz. A *t* test with $p\,\hbox{value} = 0.001$ indicates a statistical difference between the means. However, the reported ES ($d=0.0903$, $U_3=53\%$) suggests that the difference is small from a practical point of view to the extent that it does not matter which frequencies will be used to catch the user’s attention.

**Table 1:** *P* values of the *t* test between pair of conditions (without compensation for multiple comparisons) and *d*-Cohen, $U_3$-Cohen of ES for location, frequency, and contrast setups. Statistical significant results (for the *t* test and ES both) are in bold.

Frequency				Location				Contrast			
Pair	*p*	*d*	$U_3$	Pair	*p*	*d*	$U_3$	Pair	*p*	*d*	$U_3$
**1.5 Hz** $\leftrightarrow$ **2 Hz**	**0.000**	**0.3292**	**63%**	**Diag **$\leftrightarrow$ **Vert**	**0.000**	**0.391**	**65%**	**Low** $\leftrightarrow$** Med**	**0.000**	**0.44**	**67%**
**1.5 Hz** $\leftrightarrow$ **4 Hz**	**0.000**	**0.4243**	**66%**	**Diag** $\leftrightarrow$ **Horiz**	**0.000**	**0.414**	**65%**	**Low** $\leftrightarrow$ **High**	**0.000**	**0.48**	**68%**
2 Hz $\leftrightarrow$ 4 Hz	0.001	0.0903	53%	Vert $\leftrightarrow$ Horiz	1.000	0.022	51%	Med $\leftrightarrow$ High	0.851	0.03	51%

Effect size indicates that the sample probe was sufficient to differentiate the compared groups of locations (Horizontal and Vertical vs. Diagonal) (0.391–0.414) and frequencies (2 Hz and 4 Hz vs. 1.5 Hz), except the Vertical $\leftrightarrow$ Horizontal and 2 Hz $\leftrightarrow$ 4 Hz groups, where the *d* was small (equal to 0.022 and 0.09, respectively). The result suggests that the sample probe was too small, or the groups are similarly visible to the users. Taking into account the *t* test result for location ($p \,\hbox{value} = 1.000$), we suspect a similarity in visibility. For frequencies, the effect size (*d*-Cohen) is smaller than 0.2, which according to Cohen rules^52^ means that a difference exists but is too small to have practical implications.

**Figure 7:**
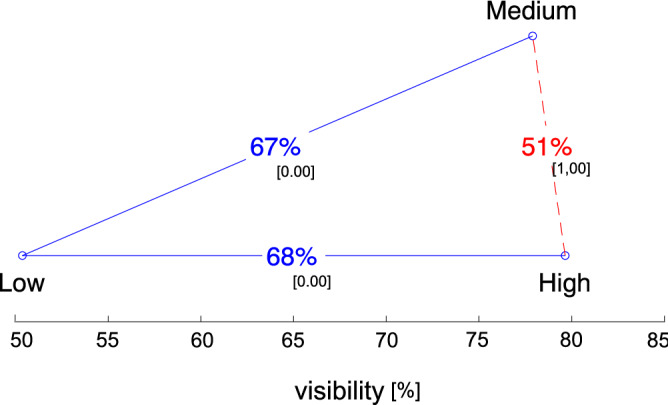
Average visibility results. The ranking shows the relationship between the groups with respect to contrast. Each blue circle represents a tested condition and they are ordered according to their ranking, with the least preferred condition on the left. The x-axis represents the rating of each condition, expressed as the mean number of votes, i. e. mean visibility of the disks with given setup. The percentages indicate the probability that an average observer will regard the condition on the right as better than the condition on the left. If the line connecting two conditions are red and dashed, there is no statistical difference between this pair of conditions ($H_0$ could not be rejected at 0.05 level). The small-font number in square brackets indicates the *p* value of the *t* test between both conditions (without compensation for multiple comparisons).

Going deeper into analyses, we checked the relations between three different contrast levels: low (1.4:1), medium (7.6:1), and high (19:1) (see Fig. 7 and Table 1). As before, one-way ANOVA for the setup was performed first. As the results pointed significant differences between the conditions ($p\,\hbox{value} = 1.931e^{-82}$, $F(2) = 197.57$), to confirm where the differences occurred, we ran posthoc tests. The most visible were discs with a high or medium contrast in relation to the background on which they were displayed. Setups of such conditions were spotted similarly ($p \,\hbox{value} = 0.851$); this means that using a medium or high contrast without taking into account the frequency or location does not change the visibility of the elements. In the case of a low contrast, it was statistically different from the two previous conditions (*p* value equal to 0.000). Effect size results show that the invited group of users was sufficient to distinguish the visibility between pairs of contrasts: Medium $\leftrightarrow$ Low and High $\leftrightarrow$ Low, with a *d*-Cohen value between 0.44 and 0.48 (see Table 1). The practical impact of the difference according to Cohen’s roles^52^ is medium; in that case, it means that almost 7 out of 10 observers saw the colored discs.

At the end, we checked if the size of the effect (i.e., the practical result) differed significantly between individual settings. In other words, does one of the settings have a more significant impact on visibility in the peripheral area than the others? Therefore, we ran a three-way ANOVA with no repeated measures (contrast $\times$ location $\times$ frequency) on the computed *d*-Cohen values; however, we did not find any statistical difference in any of the three setups (frequency, location, and contrast): F(6) $=$ 0.04, $p = 0.96$. Neither setup has higher importance than the others.

#### Two factor-setup analysis

The next step was the analysis of dependence between visibility and pairs of conditions from different setups. First, two-way ANOVA with no repeated measures was computed for contrast $\times$ location and contrast $\times$ frequency setups. In both cases, the test showed the overall difference between the setups. However, for the first one, $F$-stats was quite high ($F = 15.73$, $p\,\hbox{value} = 8.913e^{-13}$); for the second case, $F$-stats was smaller and equal to 2.4 ($p\,\hbox{value} = 0.0483$).

**Figure 8:**
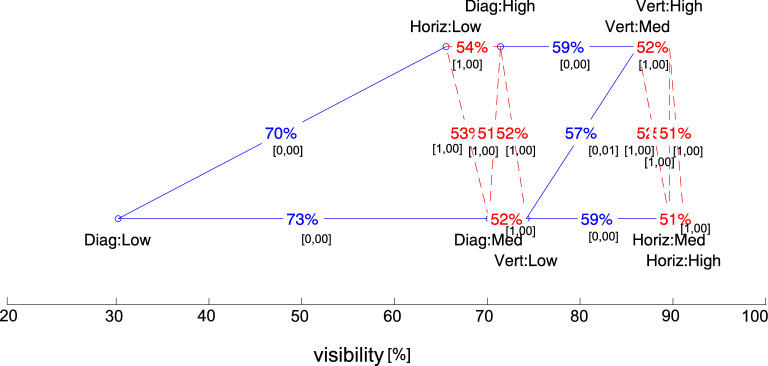
Effect size and ANOVA results: Ranking chart showing the relationship between setups in terms of contrast and disc location.

**Figure 9:**
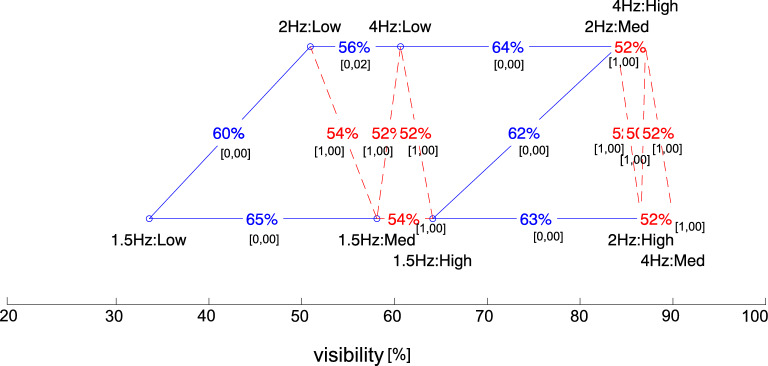
Effect size and ANOVA results: Ranking chart showing the relationship between setups in terms of contrast and flickering frequency.

Going deeper into analysis, we ran posthoc tests. We computed the *t* test between the pairs of frequencies for every contrast level: 1.5 Hz:Low $\leftrightarrow$ 2 Hz:Low, 2 Hz:Low $\leftrightarrow$ 4 Hz:Low, 1.5 Hz:Med $\leftrightarrow$ 2 Hz:Med, 2 Hz:Med $\leftrightarrow$ 4 Hz:Med, 1.5 Hz:High $\leftrightarrow$ 2 Hz:High, and 2 Hz:High $\leftrightarrow$ 4 Hz:High. We also computed the *t* test between the pairs of locations for every contrast level: Vert:Low $\leftrightarrow$ Horiz:Low, Horiz:Low $\leftrightarrow$ Diag:Low, Vert:Med $\leftrightarrow$ Horiz:Med, Horiz:Med $\leftrightarrow$ Diag:Med, Vert:High $\leftrightarrow$ Horiz:High, and Horiz:High $\leftrightarrow$ Diag:High. The results for all frequency levels and all the angular distances for each of the three contrast levels are depicted in Figs. 8 and 9. The detailed data are included in Table 2.

**Table 2:** *P* values of the *t* test between pair of conditions (without compensation for multiple comparisons) and $d$-Cohen, $U_3$-Cohen of ES for (location $\leftrightarrow$ contrast) and (frequency $\leftrightarrow$ contrast) pairs of conditions, respectively. Statistical significant results (for *t* test and ES both) are in bold.

Frequency$\leftrightarrow$ contrast				Location$\leftrightarrow$ contrast			
Pair	*p*	*d*	$U_3$	Pair	*p*	*d*	$U_3$
**Diag:Low** $\leftrightarrow$ **Horiz:Low**	**0.000**	**0.535**	**70%**	**1.5 Hz:Low** $\leftrightarrow$ **2 Hz:Low**	**0.000**	**0.252**	**60%**
**Diag:Low** $\leftrightarrow$ **Diag:Med**	**0.000**	**0.639**	**73%**	**1.5 Hz:Low ** $\leftrightarrow$ **1.5 Hz:Med**	**0.000**	**0.359**	**65%**
Horiz:Low $\leftrightarrow$ Diag:Med	1.000	0.069	53%	2 Hz:Low $\leftrightarrow$ 1.5 Hz:Med	0.489	0.102	54%
Horiz:Low $\leftrightarrow$ Diag:High	1.000	0.088	54%	2 Hz:Low $\leftrightarrow$ 4 Hz:Low	0.029	0.139	56%
Diag:Med $\leftrightarrow$ Diag:High	1.000	0.019	51%	1.5 Hz:Med $\leftrightarrow$ 4 Hz:Low	1.000	0.036	52%
Diag:Med $\leftrightarrow$ Vert:Low	1.000	0.062	52%	1.5 Hz:Med $\leftrightarrow$ 1.5 Hz:High	1.000	0.087	54%
Diag:High $\leftrightarrow$ Vert:Low	1.000	0.043	52%	4 Hz:Low $\leftrightarrow$ 1.5 Hz:High	0.000	0.051	52%
**Diag:High** $\leftrightarrow$ **Vert:Med**	**0.000**	**0.272**	**59%**	**4 Hz:Low** $\leftrightarrow$ **2 Hz:Med**	**0.000**	**0.382**	**64**%
**Vert:Low** $\leftrightarrow$ **Vert:Med**	**0.010**	**0.209**	**57%**	**1.5 Hz:High** $\leftrightarrow$ **2 Hz:Med**	**0.000**	**0.329**	**62%**
**Vert:Low**$\leftrightarrow$ **Horiz:Med**	**0.001**	**0.279**	**59%**	**1.5 Hz:High** $\leftrightarrow$ **2 Hz:High**	**0.000**	**0.381**	**63%**
Vert:Med $\leftrightarrow$ Horiz:Med	1.000	0.070	52%	2 Hz:Med $\leftrightarrow$ 2 Hz:High	1.000	0.051	51%
Vert:Med $\leftrightarrow$ Vert:High	1.000	0.064	52%	2 Hz:Med $\leftrightarrow$ 4 Hz:High	1.000	0.060	52%
Horiz:Med $\leftrightarrow$ Vert:High	1.000	0.068	52%	2 Hz:High $\leftrightarrow$ 4 Hz:High	1.000	0.010	50%
Horiz:Med $\leftrightarrow$ Horiz:High	1.000	0.000	51%	2 Hz:High $\leftrightarrow$ 4 Hz:Med	1.000	0.076	52%
Vert:High $\leftrightarrow$ Horiz:High	1.000	0.035	51%	4 Hz:High $\leftrightarrow$ 4 Hz:Med	1.000	0.067	52%

In the case of angular distances, the least visible elements were located in the corners of the display, especially in the low contrast setup. Only about 3 out of 10 users saw them ($Cohen-U_3$ was in the level 70--73%). The results are significantly different from the rest of the setup condition combinations with $p\,\hbox{value} = 0.000$. The increase in the contrast level significantly improved the visibility of these elements, but still not at a satisfactory level. Visibility in that group was still statistically worse than the discs located nearer to the center of the screen, i.e., vertically and horizontally. Additionally, with the colors corresponding to a medium and high contrast: about 6 out of 10 users saw them. The effect size of the setups was concededly small but noticeable (*d*-Cohen in the range of 0.209 to 0.279).

The levels of frequencies joined with contrast were perceived similarly to the location setup. The low flashing frequencies (1.5 Hz) and low contrast turned out to be the setup where disc visibility was the worst. This setup was perceived as the worst, at about 60–65% (with *d*-Cohen about 0.252–0.0.359 and $p\,\hbox{value} = 0.000$) compared to the others. The visibility at the frequency of 2 Hz and 4 Hz and with a medium or high contrast was so similar that it is difficult to suggest the best solution ($p\,\hbox{value} = 1.000$ and *d*-Cohen about 0.06). However, the worst setups differ significantly ($p\,\hbox{value}=0.000$), with the effect size at the small/medium level (*d*-Cohen about 0.36).

The effect size results were the most interesting from the user interface perspective. They indicate the setup compositions that had a noticeable impact on enhancing the visibility in the peripheral area. On the other hand, for the combinations that differ significantly from the rest, the test confirmed that the number of samples used in the analyses to notice the essential differences was sufficient.

As the contrast level is crucial in graphical component design, the setup combination without contrast was not more comprehensively considered.

### Habituation effect

The critical difference between sensitivity to a stimulus and attention selection or endogenous attention is the type of stimulus. Some situations make attention selective (in other words, adapted to the stimulus), and the user decides what to look at or react to and what to ignore. An example of such a stimulus is, for example, an advertisement that may be irritating to a human being. In such a situation, a person displaces an ad from his memory or ignores it with his eyesight because he/she is used to it. Another type of human focus on a stimulus is sensitivity to that stimulus. An example of such a situation is the control panel in a car’s cockpit. The person getting into the car can see the entire control panel. However, attention is paid to this only when there is an indicator, which usually lights up in orange or red. This is called tenderness or sensitization to a stimulus.

In our experiment, we focus on, among others, sensitization to the stimulus by displaying discs in different contrasts and with different frequencies at which such discs flicker. Moreover, no stimulus was displayed with the same combination (contrast level and flicker frequency) more than once. In the case of location, the disc orientation was repeated two (vertical and horizontal locations) or four times (corner locations). Thus, adaptation and endogenous attention to the stimulus during our experiment could not occur. Further analysis focused on habituation took into account results based on the parameters acceptable for practical usage with at least 80% visibility, including a high and medium contrast and a flickering frequency of at least 2 Hz. The conducted research showed the occurrence of habituation more often than sensitization. However, they were identified only for setups when the visibility was under the practical usage, i.e., below 80% (see Table 3).

Table 3 is a summary of the habituation and sensitization of observers concerning the disc depending on the contrast, the flashing frequency, and the angular distance. The habituation effect was identified when the number of positive reactions (represented by visibility) to the repeated stimuli was dropped with each repetition. In contrast, the sensitization effect was observed when attention was increased and repetitions occurred . We reported habituation/sensitization changes only above the level of changes equal to 10%. In other cases, the changes were so small that they are marked with a ’no change’ status. In Table 3, we indicate them with a gray arrow.

**Table 3:** The collective representation of habituation and sensitization for high, medium, and low contrasts, considering the flashing disc’s frequency (in Hz) and different angular distances (in degrees). A red arrow represents habituation; a blue arrow represents sensitization, and a gray arrow represents neutral to low habituation. Changes between the first (vF: first stimuli for the setup, given, given in %) and the last (vL: last stimuli for the setup, given in %) stimulus display are shown in the ch (change) column given in %, while the average value of the disc visibility in the m column (mean) is given in %. The setups with a habituation/sensitization effect with a change above 10% are marked with bold font.

	Frequency of the disc									
**High contrast**										
*Angular distance*	2 Hz	ch [%]	vF [%]	vL [%]	m [%]	4 Hz	ch [%]	vF [%]	vL [%]	m [%]
Vertical (46°)	$\rightarrow$	7.00	93	100	96	$\rightarrow$	2.04	98	96	97
Horizontal (70°)	$\rightarrow$	6.45	87	93	90	$\rightarrow$	2.20	91	89	90
**Diagonal (80°)**	$\nearrow$	**28.72**	**67**	**94**	**80**	$\rightarrow$	3.61	83	80	81
** Medium contrast**										
*Angular distance*	2 Hz	ch [%]	vF [%]	vL [%]	m [%]	4 Hz	ch [%]	vF [%]	vL [%]	m [%]
Vertical (46°)	$\rightarrow$	5.10	93	98	95	$\rightarrow$	3.89	93	94	93
Horizontal (70°)	$\rightarrow$	9.57	85	94	89	$\rightarrow$	2.08	94	96	95
Diagonal (80°)	$\rightarrow$	8.5	80	73	75	$\rightarrow$	8.89	82	90	86
** Low contrast**										
*Angular distance*	2 Hz	ch [%]	vF [%]	vL [%]	m [%]	4 Hz	ch [%]	vF [%]	vL [%]	m [%]
**Vertical (46°)**	$\searrow$	**12.67**	**71**	**62**	**66**	$\searrow$	**11.24**	**89**	**79**	**83**
**Horizontal (70°)**	$\searrow$	**12.50**	**80**	**70**	**75**	$\searrow$	**11.96**	**92**	**81**	**86**

Based on an analysis of the results,the habituation effect appears at low contrast only.When the contrast level increased to a high level, a flashing frequency of 2 Hz caused sensitization to stimuli (diagonal location). In other words, with a higher contrast level and smaller distances from the screen center (vertical and horizontal), the habituation effect seems small. At frequencies below the threshold of 2 Hz, discs were less noticeable and therefore ignored.

In the case of the location of the disc in the corners of the screen (diagonal), the sensitization effect appeared with a high contrast. Lowering the contrast to medium causes a change of 8.5%, which means that the visibility was at a similar level, independent of repetitions (in the diagonal location, we had four repetitions, i.e., four display corners). However, even for 2 Hz and 4 Hz, the visibility of the disc was lower than in the case of locations closer to the center of the screen. Therefore, we do not recommend that location, especially for critical alert cases.

*Summary.* Summarizing the results, an interesting observation is that a constant maintaining of attention was characteristic of settings with medium and high contrasts and with frequencies of 2 Hz and 4 Hz. Considering the random nature of the display of stimuli during the experiment of different intensities, the obtained result seems to be promising, classifying the indicated settings as attracting attention despite the passage of time and the cognitive task performed. The result is interesting, as attempts to consciously redirect one’s eyesight to stimuli were immediately identified by the eye-tracker and rejected from the analysis. Therefore, the data included in the analysis concern only peripheral vision. Additionally, the eye-tracker data confirmed the visual involvement in the cognitive tasks performed.

## Discussion

The main aim of the presented study was to search for effective ways of increasing the visibility of visual objects in the peripheral area without having a negative impact on the user’s task. Searching for effective communication with the use of a peripheral area was initially explored in the field of calm technologies^53^, ambient information systems^54^, peripheral displays^55^, and recently peripheral interactions^56^. Theoretical background was based on the sensitivity of sensors such as rods and cones^57^ and the impact of contrast and frequencies on user response; however, in our investigations, we focused on conscious processing rather than sensors and adaptation. That is why, in the experiment, cognitive tasks were used. We aimed for a user to concentrate on the primary task and the system’s ability to deliver the signal in the periphery area without attracting the user’s attention to peripheral stimuli. This made it possible to avoid interruptions of cognitive processes and the negative effects of task interruptions as was reported in earlier studies^58,59^. Areas of application include games, virtual reality, critical alerts and notifications presented in visual form^44^, medical systems^60^, automotive devices^61^, and other interfaces.

Notifications presented in the peripheral area do not require high cognitive loads and have the potential to be a part of multitasking without any negative impact on primary tasks within a user’s central vision. The lower intensity of peripheral stimuli makes it possible to remain engaged in a primary task while a peripheral signal is delivered, and minimal conscious control is needed^62^.However, as we discussed in the paper, not all peripheral signals have the same power to catch the user’s attention. The main set of parameters in our experiment included flashing frequency, contrast, and distance from the central area, and this set was verified.We excluded in our investigations an analysis of the deteriorated visual acuity, i.e., resolution. According to^63^, a peripheral area can be used for general signal and notice delivery but not for detailed information due to visual acuity and a sampling-limited mechanism.

An analysis of results showed that some combinations of parameters could not lead to visibility at the level of 80%, which means that more than 20% users did not notice stimuli, which is not acceptable for critical systems. The analysis that focused on habituation showed that parameters acceptable for practical usage (with at least 80% visibility) include high or medium contrast and a flashing frequency of at least 2 Hz. We confirmed the results by employing such combinations of parameters with minimal levels (providing low cognitive load), and it was possible to maintain attention on the task being performed. This is in line with earlier studies that minimal cognitive load is needed to deliver a message in peripheral areas^62^.

Earlier discussions about attention guiding techniques using peripheral vision showed the need to display objects that do not require a user to focus on them, but he/she is able to perceive their information^64^. Research has shown that the interpolation of the flickering frequency between 1 and 5 Hz makes it noticeable but not obtrusive^64^. One of the approaches is based on the modulation of luminance with a constant low frequency of 1–3 Hz^65^. Apart from a lower rate, 1–3 Hz attempts are done to use an alternative technique such as a high-frequency flicker^65^ or a decaying flicker^66^. High contrast can also improve the performance of polymorphic warnings^67^.

However, while working with user interfaces, the most desirable situation would be to increase the user’s attention to the stimuli displayed in peripheral areas, regardless of how long he/she has been working with the system, which means triggering an effect of sensitization. For rapid usage with short user sessions, we recommend components with high contrast and a 4 Hz flickering frequency located vertically or horizontally, as they are characterized by a high visibility level. Our results are consistent with Campbell et al.^61^, who stated that the attention-capturing properties of the visual warning should be maximized by having it appear abruptly within the relevant field of view and possibly by making it flash at a rate of 4 Hz.

However, longer usage can result in negative side effects, such as irritation, perceived intrusiveness, and habituation, in addition to what has been presented. In the case of long-term applications such as video sessions, games, and medical systems, a medium contrast level and a lower flickering frequency of 2 Hz seems sufficient. There is no need to increase distraction when a moderate visual intensity is enough to attract user attention. Exceeding the level of 4 Hz, from a medical point of view, can cause malaise and headaches, and even distortions, epilepsy, and perceived intrusiveness. However, while a critical alert is required, the flashing frequency should be increased to 4 Hz, where sensitization is crucial. The research in this paper is practical both from the point of view of angular distances relating to the standard distance of the user from the screen and of flashing frequencies that are acceptable in terms of the user’s health and well-being. This research may be used to create graphical interfaces that will make better use of peripheral areas (e.g., for information or alerts).

While efforts to reduce the habituation effect can increase perceived intrusiveness^37^, our priority was to avoid the habituation effect and to find the parameter setup that interrupts the user’s cognitive process as little as possible, i.e., that is minimally invasive. We reported a change in habituation/sensitization only above the level of 10%. In other cases, the changes were so small that they were marked with a ’no change’ status. We found that the habituation effect appears at low contrast only. A high contrast level and a flashing frequency of 2 Hz caused sensitization to stimuli (diagonal location). In other words, at higher contrast levels and smaller distances from the screen center (vertical and horizontal), the habituation effect seems small. At frequencies below the threshold of 2 Hz, the discs were less noticeable and therefore ignored.

Other practical implications extend current accessibility guides such as WCAG^68^. They are based on contrasts of static texts targeted mainly to texts and web design. Our study shows how contrasts can be used within flickering images to improve content visibility without negative impacts on users and distractions from primary tasks.

The presented research suggests several directions for future studies. These include adding motions to objects with a flickering effect. While motion is considered another effective way to increase awareness^69^, the target can be to minimize side effects, such as distraction and irritation. Another possible option is to integrate theoretical findings into real systems based on critical warnings and verify performance in a production setup.

## Methods

The study presented in the paper was conducted according to the guidelines of the Declaration of Helsinki, and has been approved by Bioethics Committee Agreement no KB-0012/24/2020, Bioethical Commission of Pomeranian Medical University, Szczecin - 09.03.2020. All analyzed data were fully anonymized.

### Conceptual framework

The research presented in this paper focuses on habituation and sensitization in the perception of components used in graphical interfaces within the peripheral area. Interface designers tend to catch the user’s attention, but this can, unfortunately, have an effect that is the opposite of what was intended. In other words, messages that should draw the user’s attention become intrusive in reception. By intrusiveness, we mean Li et al.’s definition, understood as a perception or psychological consequence that occurs when a person’s cognitive processes are interrupted^70^.

Our goal was to determine what disc (our stimulus) parameter values help the disc catch users’ attention in a positive manner (without irritation), together with reduced habituation, and without increasing visual intensity. Such simplified stimuli were chosen as test stimuli in the experiment because of their simplicity. They allowed us to manipulate with the parameters such as contrast, frequency, and localization. It is assumed that habituation as a behavioral response decrement will be observed as a result of repeated stimulation^8^. Another goal was to observe the effect of visual parameters on sensitization as a component of the dual process, integrating habituation with sensitization^30^. Habituation affects user interfaces and online platforms when delivery messages target users and they do not notice them. Avoiding over-exposure and negative influences on user experience, searching for the lowest possible stimuli, can overcome this effect.

**Figure 10:**
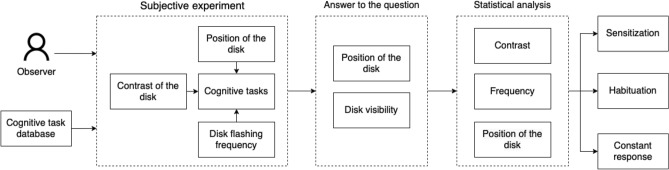
The conception overview: the process of gathering knowledge during the experiment.

The conceptual framework overview is depicted in Fig. 10. To gather the knowledge about graphical interface perception in the peripheral area, four inputs should be defined: cognitive task, the contrast of the disc color and its background, disc flashing frequency, and disc position. The data acquisition, processing, and analysis pipeline consist of three main stages: the subjective experiment, the answer to the question, and statistical analysis. As a result, sensitization, habituation or constant response outputs are returned.

Below, to resolve doubts, we explain the terminology used.

#### Cognitive task database

The cognitive task database consisted of 128 images. Each image contained a cognitive task with possible answers listed below.

#### Cognitive task

**Figure 11:**
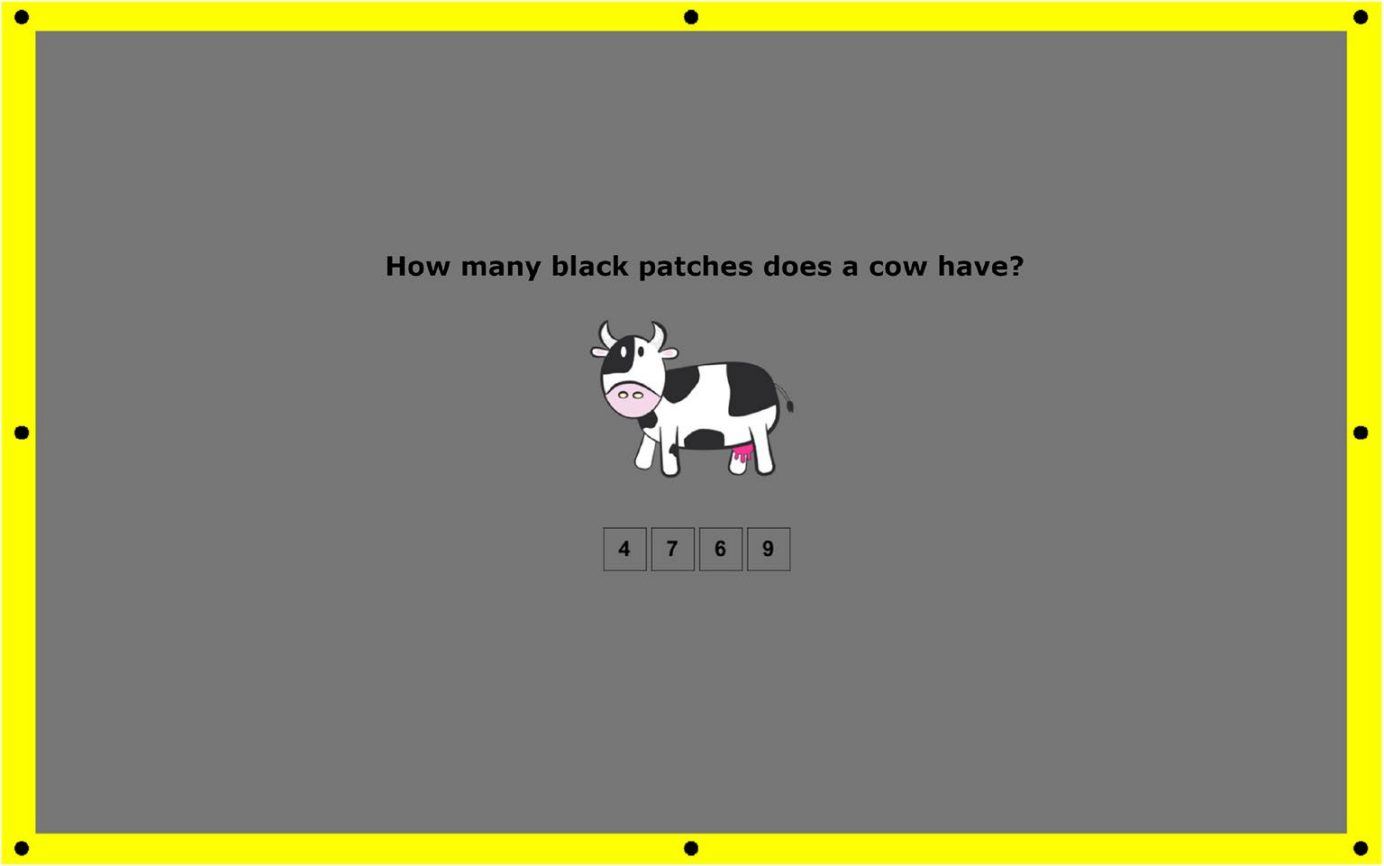
The example of the cognitive task used in our experiment. Central area (center of the screen): a cognitive task focusing the user’s attention and a box task with possible answers below. Yellow frame: an example of a colored frame that states a background for displayed discs. Elements placed on the frame (given in black color): stimuli in the form of discs, flickering with a frequency level of 1.5 Hz, 2 Hz, or 4 Hz.

The task was based on counting the elements in the image that were related to the question and choosing the correct answer (see Fig. 11).

#### Subjective experiment

The experiment was conducted with users. Every cognitive task was set up with a certain level of contrast between the color of the disc and that of the background, a specific disc flicker frequency, and a particular disc position.

#### Disk contrast

The disc was displayed with three different contrast levels computed according to Eq. (1): 1.4:1 (Blue-Violet), 7.6:1 (Black-Gray), and 19:1 (Black-Yellow). For simplicity, we refer to them as *low contrast*, *medium contrast*, and *high contrast*. This naming is consistent with the WCAG 2.0 standard^71^ recommendation. The standard covers a wide range of recommendations for making Web content more accessible. In WCAG 2.0, a contrast level choice is recommended to achieve enough contrast between the text and background to be read by people with moderately low vision (who do not use contrast-enhancing assistive technology). As color deficiencies can affect luminance contrast (C), in the recommendation, the contrast according to Eq. (1) is such that color is not a key factor, so people who have a color vision deficit will also perceive an adequate contrast between the text and the background.

1 $$\begin{aligned} C = (L_1+0.05)/(L_2+0.05) \end{aligned}$$

where $L_1$ is the relative luminance of the bright color, and $L_2$ is the relative luminance of the dark color.

A contrast ratio of 3:1 is the minimum level recommended by [ISO-9241-3] and [ANSI-HFES-100-1988] for standard text and vision. The corresponding contrast level for websites is recommended as a 4.5:1 ratio to account for the loss in contrast that results from moderately low visual acuity, congenital or acquired color deficiencies, or the loss of contrast sensitivity that typically accompanies aging. Moreover, as contrast perception decreases as the viewing angle increases, the contrast level in peripheral areas should be increased to 7:1^4^. Therefore, as a medium contrast for the experiment, we employed a 7.6:1 level, which generally provides compensation for the loss in contrast sensitivity experienced by users with low vision who do not use assisting technology and provides contrast enhancement for color deficiencies. The WCAG 2.0 standard states that a text part of a non-active graphical user interface has no minimum contrast requirement. Therefore, to adequately and clearly explain the relationship between contrast and the perception of stimulus in the peripheral areas, for a small contrast level, we chose a value below the recommended WCAG 2.0 minimum level (4.5) and established it at an F 1.4:1 level (violet-blue). As an opposite level, a high contrast was chosen that is much higher than the middle one and established at a 19.6:1 level (black-yellow).

#### Disk flicker frequency

For the design of the experiment, we took into account three levels of disc flicker frequencies: 1.5 Hz: Low, 2 Hz: Medium, and 4 Hz: High. For choosing the frequency values, we used the setup presented in^11^.

#### Position of the disc

**Figure 12:**
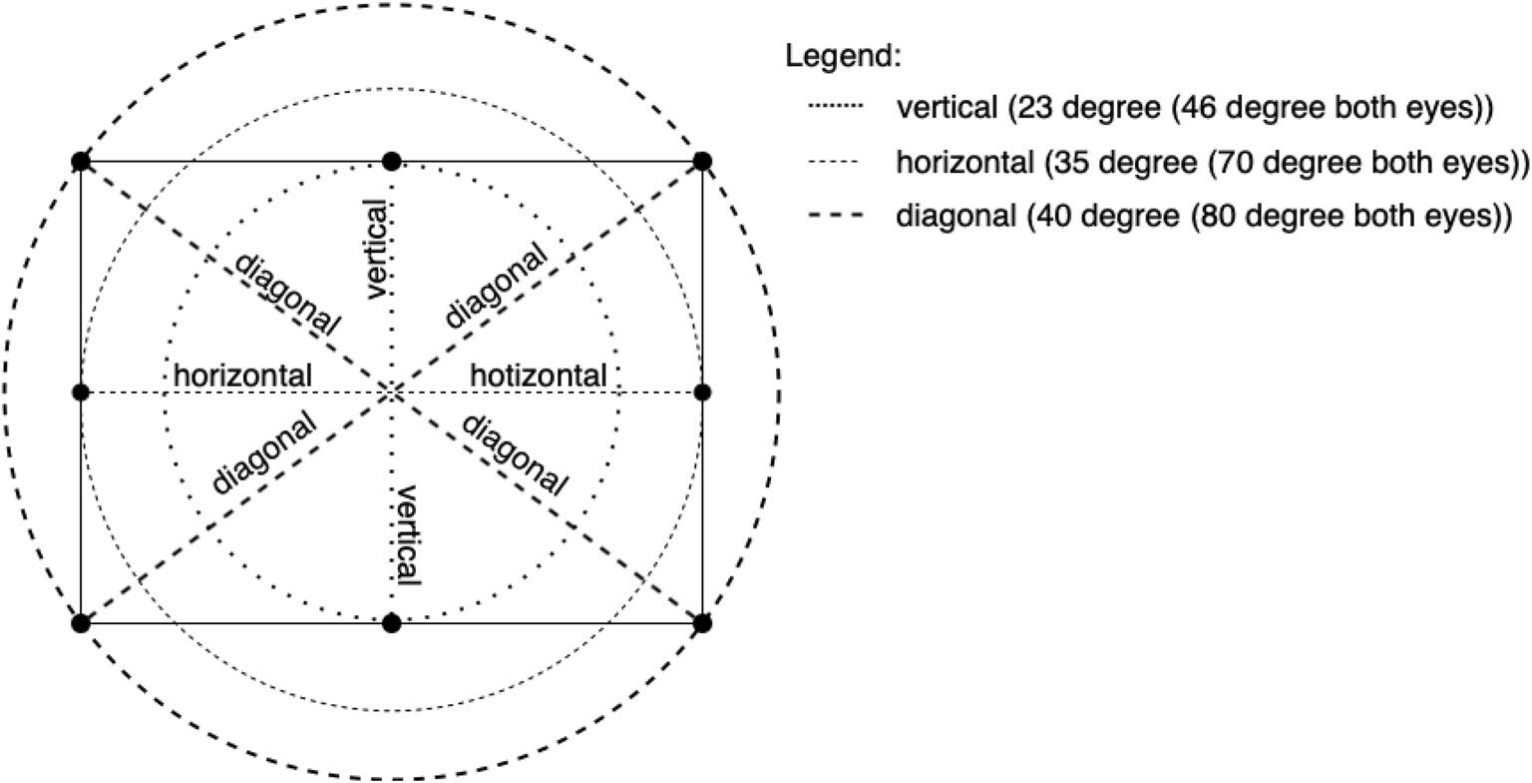
Position of the disc: The solid line that forms the rectangle is the outline of the screen on which the experiment was displayed. Black dots are discs displayed with varying frequency and contrast variations. Dashed lines represent specific angular distances (in degrees) for the following degrees of angle binocular vision (disc position): vertical (46° ($23 \times 2$)) for the middle of the bottom and top edges of the screen, horizontal (70° ($35 \times 2$)) for the middle of right and left edges of the screen, and diagonal (80° ($40 \times 2$)) for the four corners of the screen.

The localization of the disc on the screen (given in degrees of view angle) was selected based on the natural peripheral vision of humans. Because the recommended distance of a human from the monitor is 42–70 cm (in our case, 70 cm), the viewing angles assuming binocular vision had the following values: vertical (46° ($23 \times 2$)) for the middle bottom and top edge of the screen, horizontal (70° ($35 \times 2$)) for the center-right and the left edge of the screen, and diagonal (80° ($40 \times 2$)) for the four corners of the screen (see Fig. 12).

#### Answer to the question

To draw the observer’s attention to the central part of the screen, the cognitive tasks were displayed in the center of the monitor. Users had to resolve the task by choosing the correct answer (displayed below the task).

#### Answer to the question about the disc visibility

To avoiding misunderstanding, we clarify the term ***visibility***, which we define here by the percentage of users among all participants noticing the displayed discs. After each task, the observer was asked if he/she had seen the disc. The next task was displayed if the observer replied that he/she had not seen the disc. If an observer affirmed that he/she had seen the disc, the question about the location of the disc on the screen was displayed.

Finally, the statistical analysis was carried out, where the correlation between the contrast, frequency, and the location of the disc and the visibility of the disc by the observer was investigated. For this purpose, an ANOVA statistical analysis was employed. During the research, we analyzed the threshold, i.e., the lowest level, at which the elements became visible, and the habituation level, when users reacted similarly despite the number of repetitions.

### Subjective experiment

We created a consistent naming convention for all subsequent sections to avoid ambiguities. Our goal was to analyze the human perception related to the contrast level and flashing frequency of the discs displayed in the different localizations of the peripheral area of the screen. We aimed to identify when the stimulus attracts attention in a non-intrusive manner and to check for the occurrence of the habituation effect.

#### Observers

In the experiment, we examined 60 respondents who declared that they had correct vision (according to their knowledge) or that their vision had been corrected (i.e., by glasses or contacts). All analyzed data were fully anonymized. Before the experiment, the participants provided informed written consent to the use of the data collected from the perceptual experiment (according to the Bioethics Committee Agreement no KB-0012/24/2020). Computer science students, art academies students, and administration staff were invited to participate. The age of the observers ranged between 19 and 60. The average age of the respondents was 24 years. We had an additional person aged 60 and two persons aged 58 and 48. However, the standard deviation of the elderly did not differ from the entire research group. Moreover, an important aspect when designing graphical interfaces is that they cannot be adjusted to age. User interfaces are standardized regardless of the age of the people who use them^72^.

**Figure 13:**
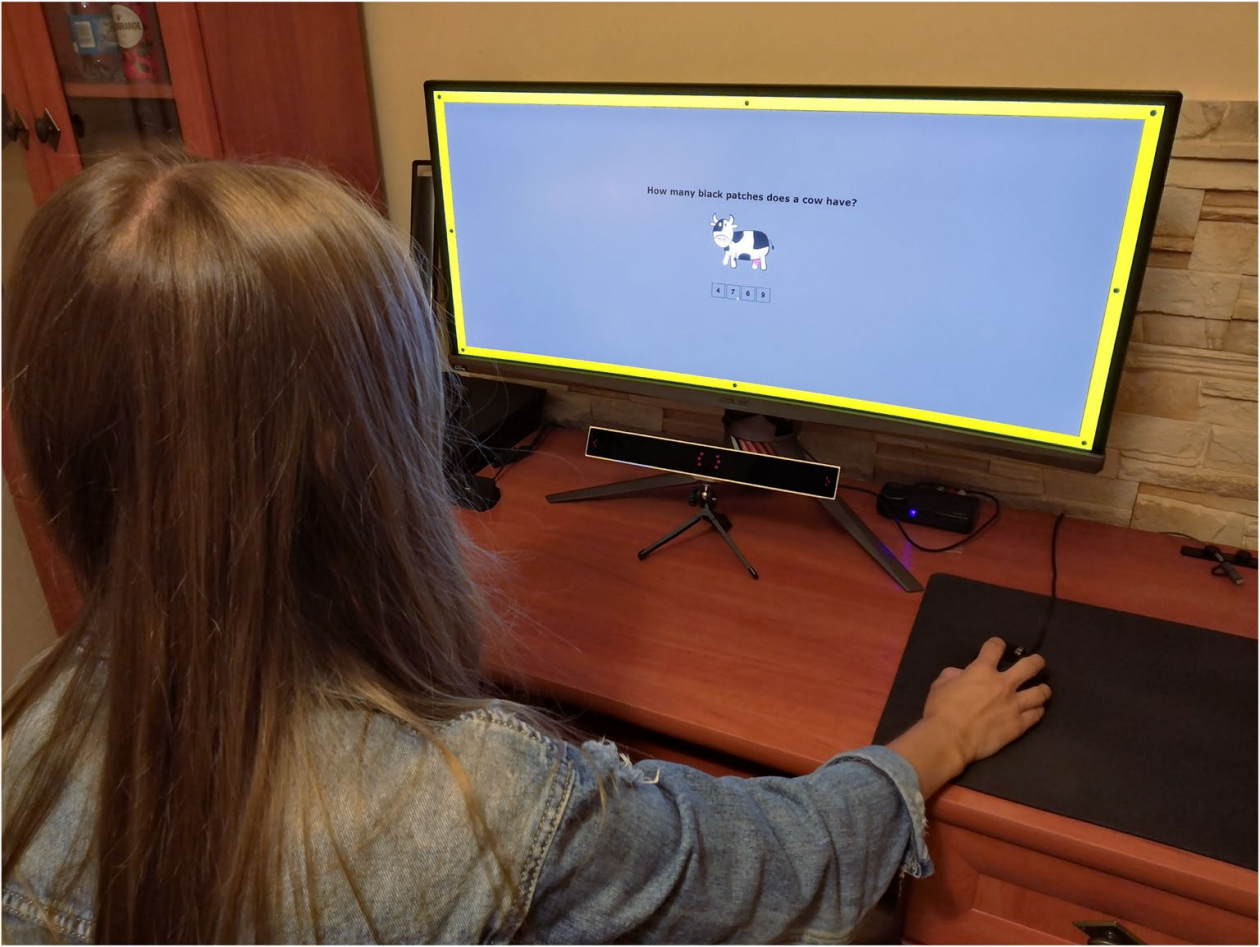
The course of the experiment and the experimental site.

#### Experimental procedure

The experiment involved investigating the visibility of the disc in the context of its display parameters. It was ensured that the disc was in the peripheral field of view. Before the experiment, observers were asked to read the written instructions. After reading the instructions, they familiarized themselves with the sample interface. Additional questions could be asked. During the experiment, we asked the users to focus on the displayed task instead of the disc because we wanted to see if a disc in the peripheral area was able to distract the user from the task at hand. We did not want the user to become accustomed to the disc so that distractions occurred naturally and were not expected.

Therefore, in the beginning, a task in the form of pictures appeared in the center of the screen, and below that, there were four possible answers. Along the edge of the screen, a frame was displayed. In that frame, eight discs were placed in selected places (positions: the upper left corner, the lower left corner, the upper right corner, the lower right corner, the center of the right edge of the screen, the center of the left edge of the screen, the center of the upper edge of the screen, and the center of the lower edge of the screen). After 2 s, one of the discs began to flash with one of three predetermined frequencies (1.5 Hz: Low, 2 Hz: Medium, and 4 Hz: High) and with one of the three preset contrasts (small, medium, and high). After 4 s, the disc stopped flashing, and the observer was obliged to perform the given task. After answering, another query was displayed: “Was there any flashing? If so, indicate which one.” If the observer answered that the disc was not flashing, another slide with a cognitive task was displayed. If the observer stated that one of the discs was flashing, the observer was asked about its position.

#### Eye tracking

The experiment was conducted with the eye-tracker Gazepoint GP3 HD 150 Hz to control, and provide confidence in, the tests’ reliability. The eye-tracker enabled us to avoid situations where the user deliberately shifted his gaze to the screen’s periphery instead of focusing on the displayed task.

Therefore, before the session, the eye-tracker calibration had to be done. There was no upper time limit for the experiment. To avoid observer fatigue, the session was prepared so that it did not last longer than 30 min. The experimental site is shown in Fig. 13.

#### Display conditions

The experiments were performed using an NEC monitor with a native resolution of $1680 \times 1050$ pixels. The monitor display was calibrated to the sRGB color space using a Minolta CS-200 colorimeter and a Specbos 1201 spectroradiometer.

#### Test images

The experiment consisted of 128 images with different cognitive tasks, e.g., requiring the counting of objects indicated in the image (see Fig. 11). The possible answers (A, B, C, and D), where one of them was correct, were placed under the task. According to^73^, perceptual experiments should last no longer than 30 min. The average experiment time was about 20–25 min, so there was no need to divide the investigation into blocks.

### Data analysis

We used the 1-way analysis of variance (ANOVA) and *effect size* (ES) statistical tests to analyze the data obtained from the experimental studies. A *t* test was employed to analyze one separated factor—frequency, location, or contrast, respectively—and to find the mean differences between pairs of factors. However, while a *p* value (returned by ANOVA) informs whether or not there is a statistically significant difference between two groups, it does not indicate how significant this difference is, opposite to the *effect size* test^74,75^.

Therefore, to emphasize and confirm the experimental results, the effect size for the difference between the compared groups with different setups (levels of contrast, flashing frequency, and angular distances) have been taken into account. A more accurate setup that easily attracts human attention should reduce randomness in visibility responses, making it more distinctive. In other words, a more proper setup should result in drawing more of the user’s attention, in a way that allows for the correct indication of a location where the flashing disc occurred under a statistical test. A simple measure of such “proper visibility” in our analysis is the ES (*d*), that is, the difference between visibility for different setups normalized by a common standard deviation:

2 $$\begin{aligned} d=\frac{|u_i-u_j|}{\sigma }. \end{aligned}$$

We computed the effect size between the pairs of frequencies for every contrast level: 1.5 Hz:Low $\leftrightarrow$ 2 Hz:Low, 2 Hz:Low $\leftrightarrow$ 4 Hz:Low, 1.5 Hz:Med $\leftrightarrow$ 2 Hz:Med, 2 Hz:Med $\leftrightarrow$ 4 Hz:Med, 1.5 Hz:High $\leftrightarrow$ 2 Hz:High, and 2 Hz:High $\leftrightarrow$ 4 Hz:High. We also computed the effect size between the pairs of locations for every contrast level: Vert:Low $\leftrightarrow$ Horiz:Low, Horiz:Low $\leftrightarrow$ Diag:Low, Vert:Med $\leftrightarrow$ Horiz:Med, Horiz:Med $\leftrightarrow$ Diag:Med, Vert:High $\leftrightarrow$ Horiz:High, and Horiz:High $\leftrightarrow$ Diag:High. These are the pairs of analyzed differences in visibility.

#### Data visualization

While a visualization of relations for pairs of conditions adjacent to each other in the ranking is easy with 3–4 conditions, with more conditions it becomes unreadable. Therefore, we presented the obtained results less traditionally, making it easier to visualize the relationship not only between the closest neighbors in the ranking but also between more distant ones. To do that, we used the graph proposed by Mantiuk et al.^76^. The method is very useful, as it not only tells whether the visibility for different setups is statistically significant (ANOVA), but also whether it is significant from a practical point of view, including ET. The results are dexicted in Fig. 6, 7 and 8.

In the graphs, the percentages shown on the relation lines are the key feature of the graphs. They represent the estimate of the probability that the setup on the right is more visible than the setup on the left. When two setups are equally visible, such probability is 50%. If one setting is always visible, the probability is 100%. The most interesting from the user interface perspective was the analysis of the ANOVA test for all frequency levels (see Fig. 9) and all angular distances (see Fig. 8) for each of the three contrast levels. The low flashing frequencies (1.5 Hz) and low contrast turned out to be the setup were disc visibility seems to be the worst. Visibility with frequencies of 2 and 4 Hz and with medium and high contrasts were so similar that it was difficult to suggest the best solution. This is one of the forms in which the effect size can be presented and, from a practical point of view, is more understandable^74,75^. If the mean visibility for both setups is $u_i$ and $u_j$, and they have a common variance $\sigma ^2$ and equal sample sizes, such probability is depicted by Eq. (3).

3 $$\begin{aligned} P = 1 - \frac{1}{\sigma \sqrt{4\pi }}\int _{-\infty }^{0}e^{\frac{-(u_i-u_j)^2}{4\sigma ^2}}dt. \end{aligned}$$

The *P* value is computed from the normal cumulative distribution function assuming that the variance of the visibility difference is $2\sigma ^2$. Such probability is very useful as it estimates the percentage of cases in which an average observer will report disc visibility in a given setting. For example, the average value of disc visibility in the vertical setting is statistically different from and better than that in the diagonal setting (see Fig. 6 Right), and the disc was more visible only by about 65% times. However, between the vertical and horizontal settings, no significant difference was found. They are visible to a similar degree (51%).

## Conclusions

Results showed that a high visual intensity did not necessarily lead to the best effects. For continuous work, a medium contrast level, a horizontal or vertical display localization, and a flashing frequency of 2 Hz are sufficient for optimal visibility in the peripheral area. In the case of critical alerts and the need for short-term intensive stimuli, it is worth highlighting these with high contrast. This configuration should be the most effective if it is not a continuous operation. This is essential, as such a setting is characterized by visibility on the same level, despite the running time. This means that the effect of habituation does not take place.

However, in situations where the work with the interface is long and tiresome (such as in medical applications), we strongly suggest avoiding high frequencies of flickering and high contrast, as they can cause unnecessary irritation or even cognitive load and thus additional fatigue during work that is already difficult. Setting the contrast at a 7:1 level and a 2 Hz flashing frequency seems to be an optimal solution. When objects were located vertically or horizontally, visibility reached 90% or higher. Moreover, similar to a more invasive setup, the visibility remained at the same level, despite the running time.
